# Dental Caries in Medicaid-Insured Preschool Children With or Without Special Health Care Needs in Northeast Ohio

**DOI:** 10.1001/jamanetworkopen.2023.0999

**Published:** 2023-02-28

**Authors:** Sarah D. Ronis, David Selvaraj, Jeffrey M. Albert, Siran M. Koroukian, Suchitra Nelson

**Affiliations:** 1 UH Rainbow Center for Child Health & Policy, Cleveland, Ohio; 2 Department of Pediatrics, Case Western Reserve University School of Medicine, Cleveland, Ohio; 3 Department of Community Dentistry, Case Western Reserve University School of Dental Medicine, Cleveland, Ohio; 4 Department of Population and Quantitative Health Sciences, Case Western Reserve University School of Medicine, Cleveland, Ohio

## Abstract

**Importance:**

Children with special health care needs (CSHCN) are recognized to be at increased risk of developing dental caries (decay). Evidence is mixed regarding the association of preventive oral health care delivered by pediatric primary care clinicians with caries experience among CSHCN.

**Objective:**

To investigate caries experience, including untreated decay, among Medicaid-enrolled preschoolers with or without special health care needs.

**Design, Setting, and Participants:**

This cross-sectional study used baseline data from the Pediatric Providers Against Cavities in Children’s Teeth study, a cluster-randomized hybrid effectiveness-implementation trial conducted among 1022 Medicaid-enrolled preschoolers aged 3 to 6 years attending well-child visits at 18 participating community pediatric primary care practices in northeast Ohio. Baseline data were collected from November 1, 2017, to August 31, 2019, with statistical analyses conducted from April to August 2022.

**Exposures:**

Presence of special health care needs, as assessed by applying the Pediatric Medical Complexity Algorithm to *International Statistical Classification of Diseases and Related Health Problems, Tenth Revision* codes, was abstracted from electronic medical records. CSHCN were defined as having either noncomplex chronic disease (presence of only 1 nonprogressive chronic condition, or multiple nonprogressive chronic conditions in a single body system) or complex chronic disease (presence of any progressive chronic condition, malignant neoplasm, or significant chronic conditions involving multiple body systems).

**Main Outcomes and Measures:**

Untreated dental decay and caries experience as assessed through clinical dental examinations using International Caries Detection and Assessment System criteria.

**Results:**

A total of 1022 children aged 3 to 6 years from 18 practices were enrolled in the study. The mean (SD) age of the study population was 4.3 (1.1) years, 554 (54.2%) were boys, and of 988 with data on race and ethnicity, 451 (45.6%) were Black. Of these, 301 of 1019 (29.5%) had a likely special health care need (225 with noncomplex chronic conditions and 76 with complex chronic conditions). The most frequent chronic conditions included asthma (n = 209) and mental or behavioral health disorders (n = 146), including attention-deficit/hyperactivity disorder, autism, and developmental delays. Overall, 296 children (29.0%) had untreated decay, and 378 (37.0%) had caries experience (decayed and filled teeth). Accounting for sociodemographic characteristics, CSHCN had 34% reduced odds of untreated decay (adjusted odds ratio [AOR], 0.66 [95% CI, 0.48-0.92]) compared with those with without chronic disease. In addition, caries experience was lower among CSHCN (AOR, 0.79 [95% CI, 0.60-1.04]).

**Conclusions and Relevance:**

In this cross-sectional study of Medicaid-enrolled children who attended well-child visits as preschoolers, untreated dental decay was lower among CSHCN compared with those without chronic conditions. This study suggests that CSHCN may have had better access to the various types of dental care facilitated in medical settings.

## Key Points

**Question:**

How do untreated caries and caries experience vary among Medicaid-enrolled preschoolers with vs without special health care needs?

**Findings:**

In this cross-sectional study of a cohort of 1022 Medicaid-enrolled preschoolers, after accounting for key covariates, children with special health care needs had reduced odds of untreated caries compared with peers without chronic conditions.

**Meaning:**

This study suggests that Medicaid-enrolled children with special health care needs may have better access than children without special health care needs to the types of oral health services provided in medical settings, including regular surveillance, preventive oral health services, and referrals to dental treatment.

## Teaser

This cross-sectional study uses data from Pediatric Providers Against Cavities in Children’s Teeth study to investigate caries experience, including untreated decay, among Medicaid-enrolled preschoolers with or without special health care needs.

## Introduction

Dental caries is widely prevalent among US children and youth, with a growing burden as children age. It is estimated that more than 1 in 5 preschoolers (21.4%) have experienced caries, increasing to more than half of school-aged children (50.5%) and adolescents (53.5%).^1^ Children with special health care needs (CSHCN—children with chronic medical, emotional, developmental, behavioral, or functional limitations requiring use of health services beyond that of typical peers^2^) are recognized to be at special risk of developing caries due to a number of differences related to care of their chronic conditions. Acknowledging heterogeneity in caries risk among the various chronic conditions contributing to special health care needs (eg, physical health conditions such as asthma or malignant neoplasm vs emotional, mental, or behavioral conditions such as attention-deficit/hyperactivity disorder [ADHD] vs developmental disorders such as autism),^3^ potential child-level mechanisms for increased caries risk among CSHCN include high sugar content in medications,^4^ altered food consumption behaviors,^5^ consumption of cariogenic foods,^6,7^ poor salivary flow,^8^ alteration by inhaled drugs of oral cavity acidity,^8,9^ and enamel defects.^10,11^ Proposed family-level risk factors for poor dental outcomes among CSHCN include competing medical needs, trade-offs in care due to family financial strain,^12^ and difficulties finding dentists willing to care for CSHCN.^13,14^ Studies using administrative data indicate that young CSHCN are significantly less likely than peers to use preventive dental services^15^ and are more likely to use dental treatment services,^16^ although these differences appear to resolve as children age.^17^

There is also evidence for disparities in access to dental services according to region where the child lives, race and ethnicity, household income and socioeconomic status, and degree of disability associated with the child’s special health care needs.^18,19^ These socioeconomic and medical factors may be associated with children’s access to insurance coverage. It is not clear from prior studies whether disparities in access to and use of dental services among CSHCN are independent of or the result of differences in access to care more generally. Even among those with insurance, disparities in dental care have been observed, wherein Medicaid-insured CSHCN have been shown to use dental care significantly less often than children without special health care needs.^15^

Given primary care clinicians’ potential role in closing gaps in preventive dental services, a clearer understanding is needed regarding various children’s risks for caries and the opportunities these clinicians have for caries prevention and/or intervention as part of medical care. The objective of this study was to investigate caries experience, including untreated decay, among Medicaid-enrolled preschoolers with and without special health care needs attending well-child visits (WCVs) in community pediatric practices.

## Methods

### Study Design

This cross-sectional study used baseline data from the Pediatric Providers Against Cavities in Children’s Teeth study, a pragmatic parallel-group cluster randomized clinical trial that aimed to assess the effectiveness of a multilevel intervention bundle to increase use of dental care among children ages 3 through 6 years enrolled in Medicaid (NCT03385629).^20^ The institutional review board of University Hospitals Cleveland Medical Center approved the study protocol. Written consent in English was obtained from clinicians and parents or caregivers. The protocol followed the Strengthening the Reporting of Observational Studies in Epidemiology (STROBE) reporting guideline for cross-sectional investigations.^21^

### Setting

Study sites included 18 community-based pediatric primary care practices in northeast Ohio. Prior to study enrollment, 14 sites (78%) were participating in a quality improvement initiative to provide preventive oral health care services including fluoride varnish application as part of WCVs for children 1 to 3 years old. Participating practices, primary care clinicians, and parent-child dyads were recruited from November 1, 2017, through August 31, 2019.

### Participants

Parent-child dyads were recruited from WCVs with participating primary care clinicians. To be included in the study, the child had to be 3 to 6 years of age at the time of study enrollment and insured by Medicaid. Their parent or caregiver (hereafter, “parent”) was required to be the child’s legal guardian, aged 18 years or older, comfortable conversing in English, and with intent to reside in the study area (northeast Ohio) throughout the study duration (≥2 years from baseline). All parent-child dyads completing the baseline visit received a $40 incentive as compensation for their time.

### Data Sources and Variables

Sociodemographic variables were collected via a baseline questionnaire completed by parent self-report at the WCV encounter from which they and their child were recruited. These variables in the questionnaire were adapted from the National Health and Nutrition Examination Survey, and items collapsed into fewer categories as follows: child and parent sex (male or female), child race (American Indian or Alaska Native, Asian, Hawaiian or Pacific Islander, White, and >1 race were collapsed to “non-Black,” while Black or African American was “Black” for analysis [races were collapsed into non-Black and Black because, with the exception of White children, sample sizes of races were too small to handle individually, and there is prior literature indicating disparities in caries experience among Black children compared with non-Black children^22^]), child ethnicity (Hispanic or non-Hispanic), child age in years, parental educational attainment (grades 7-9, grades 10 and 11, and high school diploma or General Educational Development certification were collapsed as “≤high school,” while associate’s degree or some college, college or university degree, some graduate school, and graduate degree were collapsed for analyses to “>high school”), and parental marital status (common law, divorced, separated, single, and widow were collapsed for analyses as “not married,” while “married” was a category by itself). The questionnaire also addressed oral health behaviors, including whether the child had ever visited a dentist (yes or no) and, if so, at what age (in years) the child had their first dental visit.

The presence of special health care needs was assessed based on medical records data from the 12 months preceding the baseline visit. Chronic and nonchronic conditions were abstracted from the child’s electronic medical record as *International Statistical Classification of Diseases and Related Health Problems, Tenth Revision* (*ICD-10*), codes. We applied the least-conservative form of the Pediatric Medical Complexity Algorithm (PMCA), version 3.0,^23^ to assign chronic condition status. “Noncomplex chronic disease” was defined as ever having only 1 nonprogressive chronic condition, or multiple nonprogressive chronic conditions involving a single body system. “Complex chronic disease” was defined as ever having any progressive chronic condition, any malignant neoplasm, or multiple nonprogressive chronic conditions involving multiple body systems. We interpreted children with either complex or noncomplex chronic disease as CSHCN and we defined children with no chronic disease as non-CSHCN. As data for the parent-reported consequences-oriented definition delineated by the CSHCN screener^2^ were not available, we expected that a diagnosis-based definition following the PMCA would suitably represent the range of children cared for in primary care practices serving low-income children.

The outcome variables, untreated decay (decayed teeth) and caries experience (decayed and filled teeth), were assessed through clinical dental examinations completed in the practices during the WCVs from which children were recruited. Six licensed dental hygienists were trained in the International Caries Detection and Assessment System (ICDAS). Teeth were visually inspected without magnification or radiographs after cleaning with a toothbrush and drying with gauze. International Caries Detection and Assessment System lesion codes for decay included 0 for sound teeth, 1 to 2 for noncavitated early decay, and 3 to 6 for localized to extensive cavitation. International Caries Detection and Assessment System filling codes included 0 for sound teeth, 1 to 2 for full and partial sealants, and 3 to 6 for tooth-colored restoration, amalgam restoration, and stainless steel, ceramic, gold, or porcelain crowns. Untreated decay was defined as presence of ICDAS lesion codes of 3 or more on 1 or more teeth. Caries experience was assessed by presence of either or both of ICDAS lesion codes of 3 or more and ICDAS filling codes of 3 or more on 1 or more teeth. The findings of dental hygienists were calibrated against a criterion standard dentist examination, with a weighted κ of 0.67 to 0.83 for decay and an unweighted κ of 0.85 to 0.92 for fillings, indicating good to excellent interrater reliability. For analysis, untreated decay and decayed and filled teeth were further categorized as no (untreated decay or decayed and filled teeth = 0) and yes (untreated decay or decayed and filled teeth ≥1).

### Statistical Analysis

All statistical analyses were conducted from April to August 2022 using rms, tidyverse packages in R, version 4.2.0 (R Group for Statistical Computing), RStudio, version 2022.02.3 (Build 492), and SAS, version 9.4 (SAS Institute Inc). Descriptive statistics (mean values, frequencies, and proportions) were used to characterize the sample based on sociodemographic characteristics, CSHCN and caries status, and child dental visits.

Bivariate analyses (*t* test and χ^2^ test) investigated the sociodemographic differences between children with and children without special health care needs and between those with and those without untreated decay and by caries experience. Sociodemographic variables found in bivariate analyses to be associated with untreated decay, decayed and filled teeth, and/or CSHCN status at *P* < .10 were considered for inclusion in multivariable models. A generalized estimating equations (GEE) approach, based on a multivariable logistic regression model, was used to assess the association between CSHCN status and the occurrence of untreated decay and (separately) caries experience, after controlling for selected sociodemographic variables. Clustering of children within a practice was accommodated using an exchangeable working correlation structure. Estimated model coefficients were exponentiated to provide estimated odds ratios (ORs) and tests for nonnull ORs via Wald χ^2^ tests were performed and 95% CIs for ORs computed.

Secondary analysis was performed to examine a possible dose-response association between outcomes and increasingly severe CSHCN levels: no chronic disease, noncomplex chronic disease, and complex chronic disease. A GEE approach was conducted with the same model as previously described but using a score for CSHCN status (with 0 indicating no chronic disease, 1 indicating noncomplex chronic disease, and 2 indicating complex chronic disease) separately for each outcome (untreated decay and decayed and filled teeth). Odds ratios for trend (the increase in the odds of the outcome for a 1-level increase in CSHCN severity) were estimated, Wald χ^2^ tests for nonnull ORs were performed, and 95% CIs for ORs were computed. As an additional comparison of levels of CSHCN, GEE analyses were performed using 2 indicator variables for the 3-category CSHCH status; ORs for paired comparisons among these levels were assessed.

Because missing data were rare among variables selected for inclusion, GEE models were constructed using complete cases. Statistical significance in the final multivariable models was assessed at a 2-sided *P* < .05.

## Results

A total of 1233 parents were approached for participation, of whom 209 either refused or were ineligible for the study, resulting in 1024 recruited parent-child dyads.^24^ Two children were subsequently excluded as they were later found to not meet inclusion criteria. Of the 1022 children in the enrolled sample, the mean (SD) age was 4.3 (1.1) years; 466 (45.6%) were girls, and 554 (54.2%) were boys. Of 988 with data on race and ethnicity, 451 (45.6%) were Black, and 881 (86.2%) were not Hispanic or Latino (Table 1). Among their parents, 339 of 1005 (33.7%) were married and 557 of 1006 (55.4%) had at least a high school education. Of the 1019 children with *ICD-10* data, 718 (70.5%) had no chronic disease, 225 (22.1%) had noncomplex chronic disease, and 76 (7.5%) had complex chronic disease (overall, 301 of 1019 children [29.5%] were deemed likely to have a special health care need). The most frequent chronic conditions included diseases of the respiratory system such as asthma (n = 209) and mental or behavioral health disorders (including ADHD, autism, and developmental delays [n = 146]) (Table 2).

**Table 1.** Baseline Characteristics of Patients Enrolled in the PACT Study

Variable	Overall (N = 1022)	CSHCN		*P* value	Untreated decay		*P* value
		No (n = 718)	Yes (n = 301)		No (n = 726)	Yes (n = 296)	
Child age, No. (%)							
3 y	278 (27.2)	204 (28.4)	73 (24.3)	.03^a^	215 (29.6)	63 (21.3)	<.001^a^
4 y	307 (30.1)	228 (31.8)	79 (26.2)		234 (32.3)	73 (24.7)	
5 y	231 (22.6)	156 (21.7)	75 (24.9)		136 (18.7)	95 (32.1)	
6 y	205 (20.1)	130 (18.1)	74 (24.6)		140 (19.3)	65 (22.0)	
Child race, No./total No. (%)							
Black	451/988 (45.6)	322/693 (46.5)	129/293 (44.0)	.53	305/699 (43.6)	146/289 (50.5)	.06
Non-Black^b^	537/988 (54.4)	371/693 (53.5)	164/293 (56.0)		394/699 (56.4)	143/289 (49.5)	
Child ethnicity, No. (%)							
Hispanic or Latino	76 (7.4)	45 (6.3)	31 (10.3)	.08	57 (7.9)	19 (6.4)	.71
Not Hispanic or Latino	881 (86.2)	626 (87.2)	253 (84.1)		624 (86.0)	257 (86.8)	
Prefer not to answer	65 (6.4)	47 (6.5)	17 (5.6)		45 (6.2)	20 (6.8)	
Child sex, No. (%)							
Female	466 (45.6)	355 (49.4)	110 (36.5)	.001^a^	327 (45.0)	139 (47.0)	.58
Male	554 (54.2)	362 (50.4)	191 63.5)		397 (54.7)	157 (53.0)	
Prefer not to answer	2 (0.2)	1 (0.1)	0		2 (0.3)	0	
Parent educational level, No./total No. (%)							
≤High school	449/1006 (44.6)	318/704 (45.2)	130/300 (43.3)	.64	306/713 (42.9)	143/293 (48.8)	.10
>High school	557/1006 (55.4)	386/704 (54.8)	170/300 (56.7)		407/713 (57.1)	150/293 (51.2)	
Parent marital status							
Married	339/1005 (33.7)	242/707 (34.2)	96/296 (32.4)	.63	250/712 (35.1)	89/293 (30.4)	.17
Not married	666/1005 (66.3)	465/707 (65.8)	200/296 (67.6)		462/712 (64.9)	204/293 (69.6)	
Parent employment status							
Employed	681/994 (68.5)	466/697 (66.9)	213/295 (72.2)	.11	492/707 (69.6)	189/287 (65.9)	.28
Unemployed	313/994 (31.5)	231/697 (33.1)	82/295 (27.8)		215/707 (30.4)	98/287 (34.1)	
Dental visit ever or starting at what age, mo, No./total No. (%)							
No visit	307/980 (31.3)	229/691 (33.1)	78/287 (27.2)	.02^a^	226/695 (32.5)	81/285 (28.4)	.45
0-11	71/980 (7.2)	47/691 (6.8)	24/287 (8.4)		49/695 (7.1)	22/285 (7.7)	
12-23	163/980 (16.6)	98/691 (14.2)	64/287 (22.3)		109/695 (15.7)	54/285 (18.9)	
24-35	182/980 (18.6)	129/691 (18.7)	52/287 (18.1)		134/695 (19.3)	48/285 (16.8)	
≥36	257/980 (26.2)	188/691 (27.2)	69/287 (24.0)		177/695 (25.5)	80/285 (28.1)	
Untreated decay, No. (%)							
No	726 (71.0)	495 (68.9)	228 (75.7)	.04^a^	NA	NA	NA
Yes	296 (29.0)	223 (31.1)	73 (24.3)		NA	NA	
Caries experience, No. (%)							
No	644 (63.0)	445 (62.0)	196 (65.1)	.38	NA	NA	NA
Yes	378 (37.0)	273 (38.0)	105 (34.9)		NA	NA	
No. of primary teeth, mean (SD)	19.1 (2.0)	19.3 (1.7)	18.7 (2.5)	<.001^a^	19.1 (2.1)	19.1 (1.8)	.64
Chronic disease, No./total No. (%)							
None	718/1019 (70.5)	NA	NA	NA	495/723 (68.5)	223 (75.3)	.04^a^
Noncomplex chronic disease	225/1019 (22.1)	NA	NA		166/723 (23.0)	59 (19.9)	
Complex chronic disease	76/1019 (7.5)	NA	NA		62/723 (8.6)	14 (4.7)	

Abbreviations: CSHCN, children with special health care needs; NA, not applicable; PACT, Pediatric Providers Against Cavities in Children’s Teeth.

^a^ Significant at *P* < .05.

^b^ Includes American Indian or Alaska Native, Asian, Hawaiian or Pacific Islander, White, and more than 1 race.

**Table 2.** Most Frequent Conditions Documented Among Children With Likely Special Health Care Needs Enrolled in the PACT Study, 2017-2019^a^

Condition	Children, No. (%)		
	With noncomplex chronic disease (n = 225)	With complex chronic condition (n = 76)	Total (N = 301)
Mental or behavioral disorders^b^	92 (40.9)	54 (71.1)	146 (48.5)
Diseases of the respiratory system^c^	151 (67.1)	58 (76.3)	209 (69.4)
Congenital malformations, deformations, and chromosomal abnormalities^d^	16 (7.1)	27 (35.5)	43 (14.3)
Diseases of the blood and blood-forming organs and certain disorders involving the immune mechanism^e^	36 (16.0)	21 (27.6)	57 (18.9)
Endocrine, nutritional, and metabolic diseases^f^	52 (23.1)	28 (36.8)	80 (26.6)
Other: none of the above	5 (2.2)	1 (1.3)	6 (2.0)

Abbreviation: PACT, Pediatric Providers Against Cavities in Children’s Teeth.

^a^ Chronic condition categories as defined by *International Statistical Classification of Diseases and Related Health Problems, Tenth Revision* ontology chapters. Examples provided represent most frequent specific diagnoses in study data set from each chapter.

^b^ Expressive language disorder, attention-deficit/hyperactivity disorder, specific developmental disorder of motor function, or autistic disorder.

^c^ Asthma.

^d^ Atrial septal defect.

^e^ Anemia due to glucose-6-phosphate dehydrogenase deficiency or sickle cell disease.

^f^ Disorders of iron metabolism or gastroesophageal reflux disease.

Compared with those without chronic disease, CSHCN were older, less often female, and more often had initiated dental care at younger ages. As also shown in Table 1, 378 children (37.0%) enrolled in the study had decayed and filled teeth and 296 (29.0%) had untreated decay at baseline. Children with untreated decay were significantly older than those without untreated decay. Children with untreated decay less often had special health care needs compared with those without untreated decay (73 of 301 [24.3%] vs 223 of 718 [31.1%]; *P* = .04). Furthermore, untreated decay was least prevalent among those with complex chronic disease (18.4% [14 of 76]), vs 26.2% (59 of 225) among those with noncomplex chronic disease and 31.1% (223 of 718) among those without chronic disease. There was no significant difference in decayed and filled teeth among CSHCN vs non-CSHCN (34.9% [105 of 301] vs 38.0% [273 of 718]; *P* = .38).

The final set of covariates selected for the GEE were chosen based on the bivariate analysis or as reported in the literature, and they included child age and race, as well as parental educational level (Table 3). Compared with those without chronic disease, CSHCN had 34% reduced odds of untreated decay at study baseline (adjusted odds ratio [AOR], 0.66 [95% CI, 0.48-0.92]; *P* = .02). The direction and magnitude of association between CSHCN status and decayed and filled teeth (AOR, 0.79 [95% CI, 0.60-1.04]; *P* = .10) was similar to the model for untreated decay in multivariable analysis after accounting for key covariates.

**Table 3.** Generalized Estimating Equations Model With CSHCN Status as Yes or No (N = 973)

Variable	Untreated decay		Total caries experience	
	Estimated AOR (95% CI)^a^	*P* value	Estimated AOR (95% CI)^a^	*P* value
Intercept	0.21 (0.11-0.40)	<.001^b^	0.13 (0.07-0.21)	<.001^b^
CSHCN vs no CSHCN	0.66 (0.48-0.92)	.02^b^	0.79 (0.60-1.04)	.10
Age	1.27 (1.11-1.46)	<.001^b^	1.51 (1.33-1.70)	<.001^b^
Black race vs non-Black race	0.78 (0.62-1.01)	.06	0.83 (0.62-1.11)	.20
Parental educational level >high school vs ≤high school	0.79 (0.60-1.04)	.09	0.89 (0.68-1.17)	.42

Abbreviations: AOR, adjusted odds ratio; CSHCN, children with special health care needs.

^a^ Estimated AORs with 95% CI and *P* values determined via Wald χ^2^ tests.

^b^ Significant at *P* < .05.

Evidence for a dose-response association between each caries outcome and level of CSHCN was found for untreated decay (AOR, 0.70 [95% CI, 0.54-0.92]; *P* = .01) and for decayed and filled teeth (AOR, 0.78 [95% CI, 0.64-0.94]; *P* = .009) (eTable 1 in Supplement 1). Thus, there is an estimated 30% decrease in the odds of untreated decay and 22% decrease in the odds of decayed and filled teeth for a 1-level increase in CSHCN (no disease to noncomplex chronic disease, or noncomplex to complex chronic disease). Pairwise comparisons showed statistically significant ORs for no chronic disease vs complex chronic disease for both outcomes (AOR, 0.45 [95% CI, 0.22-0.92]; *P* = .03 for untreated decay; AOR, 0.48 [95% CI, 0.29-0.80]; *P* = .004 for decayed and filled teeth), and for noncomplex vs complex chronic disease for decayed and filled teeth (AOR, 0.53 [95% CI, 0.29-0.97]; *P* = .04) (eTable 2 in Supplement 1).

## Discussion

In this study of Medicaid-enrolled preschool-aged children attending WCVs in community pediatric practices, nearly 3 in 10 (29.5%) were identified as likely to have a special health care need. Our estimate is not substantially different than that generated by the National Survey of Children’s Health (NSCH) 2019-2020, which estimates a prevalence of CSHCN among public-insured children in Ohio of 28.8% (95% CI, 22.7%-35.9%).^25^ Although the NSCH uses a parent-reported consequences-oriented definition of special health care needs rather than a diagnosis-based definition as in this study, it is likely that our study population suitably represents the range of children cared for in primary care practices serving low-income children.

The total caries experience in our cohort was high, exceeding national rates for children of similar age for both untreated decay (29.0% in our sample vs 8.8% nationally) and total caries experience (37.0% in our sample vs 21.4% nationally).^1^ Our finding of fewer caries among CSHCN was unexpected, as prior literature made a compelling case for greater disparities in dental caries among this doubly at-risk population. For example, in a recent study of nearly 24 000 Medicaid-insured children in Ohio, children with complex medical conditions accounted for nearly one-third of those with early-onset decay, incurring nearly 10-fold greater costs in care.^26^ However, the significant dose-response association in our data between prevalence of dental caries and complexity of chronic conditions, as well as the consistency of our findings across analyses, are suggestive that our finding is not spurious.

One explanation may be that, unlike in prior dental literature that focused on specific chronic conditions, such as autism, we instead opted to take a condition-agnostic approach. Our motivation for this approach was multiple: in contrast to subspecialists who focus on a particular disease or body system, practicing pediatric primary care clinicians care for a heterogeneous population of children, of whom most are healthy. In contrast, with adult populations, for whom many patients have relatively few highly prevalent chronic conditions, the subset of children in a given practice with special health care needs represents a wide range of comparatively rare conditions. This variability in the types and severity of conditions associated with increased need for health care and related services necessitates that clinicians apply noncategorical approaches to care that are equally pertinent across multiple conditions.^27,28^

### Strengths and Limitations

This study has some strengths; a major strength is that dental hygienists screened the children at the practices and collected clinical caries data. Therefore, our study is the first, to our knowledge, to prospectively assess primary caries clinical data among Medicaid-insured children attending WCVs in community practices, as opposed to secondary administrative or claims data irrespective of engagement with a family-centered medical home. In addition, the presence of a special health care need may be associated with differences in the quality and/or frequency of the child’s interaction with their primary care clinician, which in turn may have a beneficial association with the child’s experience of caries. Prior literature has shown that CSHCN attend preventive visits more often than do children without special health care needs.^29^ Best practices for management of many pediatric chronic conditions, including more prevalent conditions such as asthma and ADHD, are predicated on at least a quarterly, rather than annual, visit with their primary care clinician.^30,31^ The greater number of interval visits expected of CSHCN compared with typical preschoolers without special health care needs may be providing more frequent opportunities for pediatric primary care clinicians to screen for caries, reinforce preventive oral health care messages, apply fluoride varnish, and connect families with dental clinicians who are willing and ready to care for young Medicaid-insured CSHCN.

We found that compared with children without special health care needs, CSHCN in our sample more often reported that they had already seen a dental clinician and that they did so at younger ages. This finding was consistent with administrative data from Iowa indicating that children with chronic conditions were more likely to have an earlier first dental visit after enrolling in Medicaid,^32^ and evidence emerging from a wide range of Medicaid programs that preventive medical care (especially when preventive oral health messages are provided in medical settings) increases use of dental care.^33^

Our study does have several limitations. First, the measurement of our outcome variable required cooperation of the child during the clinical dental examination, but there were only a few instances of behavioral problems among these children, and the dental hygienists were able to handle to them. However, the dental examinations were conducted in a nontraditional setting with hygienists wearing portable headlamps, which may have contributed to some underreporting of caries. Second, most practices involved in our study were participating in quality improvement initiatives where clinicians were applying fluoride varnish in the office at WCVs starting at 1 year of age. However, this exposure would be expected to be uniform unless children without special health care needs were disproportionately missing their WCVs altogether. Third, as this study relies on cross-sectional data, we are unable to attribute causality to the association between special health care needs and caries experience. Our sample is limited to Medicaid-insured children seen in primary care practices in northeast Ohio; we are unable to apply our findings to children not attending primary care visits.

## Conclusions

Among Medicaid-enrolled children engaged in pediatric primary care, odds of untreated decay were lower among CSHCN compared with children without chronic conditions. This cross-sectional study suggests that CSHCN may have better access to various types of dental care provided in medical settings, including regular surveillance and parent education by pediatric clinicians, application of fluoride varnish to prevent caries, and connections to definitive treatment when caries arise. Pediatricians caring for Medicaid-enrolled preschoolers, with or without special health care needs, should treat every visit as an opportunity for dental screening, education, and referral to treatment services.

## Notes

Supplement 1. **eTable 1.** Generalized Estimating Equations (GEE) Model With an Ordinal Score (0, 1, 2) for CSHCN Status (n=973) **eTable 2.** Generalized Estimating Equations (GEE) Model With Two Indicator Variables for the Three-Category CSHCN Status (n=973)

Supplement 2.
Data Sharing Statement
