# Supplementary material for: Dental Caries in Medicaid-Insured Preschool Children With or Without Special Health Care Needs in Northeast Ohio
Source: JAMA Netw Open. 2023 Feb 28;6(2):e230999. doi: 10.1001/jamanetworkopen.2023.0999 (PMC9975899; doi:10.1001/jamanetworkopen.2023.0999)
Supplement: Supplement 1. — eTable 1. Generalized Estimating Equations (GEE) Model With an Ordinal Score (0, 1, 2) for CSHCN Status (n=973) eTable 2. Generalized Estimating Equations (GEE) Model With Two Indicator Variables for the Three-Category CSHCN Status (n=973) [file jamanetwopen-e230999-s001.pdf]

Supplementary Online Content

Ronis SD, Selvaraj D, Albert JM, Koroukian SM, Nelson S. Dental caries in Medicaid-insured preschool children with or without special health care needs in northeast Ohio. *JAMA Netw Open.* 2023;6(2):e230999. doi:10.1001/jamanetworkopen.2023.0999

eTable 1. Generalized Estimating Equations (GEE) Model With an Ordinal Score (0, 1, 2) for CSHCN Status (n=973)

eTable 2. Generalized Estimating Equations (GEE) Model With Two Indicator Variables for the Three-Category CSHCN Status (n=973)

This supplementary material has been provided by the authors to give readers additional information about their work.

© 2023 Ronis SD et al. *JAMA Network Open.*

eTable 1. Generalized Estimating Equations (GEE) Model With an Ordinal Score (0, 1, 2) for CSHCN Status (n=973)

	Untreated Caries (dt)†		Total Caries Experience (dft)†	
**Variables**	**Estimated AOR (95% CI)**	**p-value**	**Estimated AOR (95% CI)**	**p-value**
(intercept)	0.211 (0.111,0.403)	<0.001*	0.126 (0.074,0.216)	<0.001*
CSHCN Score	0.702 (0.536, 0.918)	0.010*	0.775 (0.639, 0.939)	0.009*
Age	1.271 (1.112, 1.457)	<0.001*	1.511 (1.339, 1.704)	<0.001*
Black Race (ref. Non Black)	0.791 (0.624, 1.002)	0.052	0.832 (0.628, 1.103)	0.202
Parent Education > High School (ref. <= HS)	0.792 (0.603, 1.041)	0.094	0.892 (0.678, 1.173)	0.413

†Estimated Odds Ratios with 95% Confidence Interval (CI) and p-values via Wald Chi-Square Tests

***Significance at p<0.05**

© 2023 Ronis SD et al. *JAMA Network Open.*

eTable 2. Generalized Estimating Equations (GEE) Model With Two Indicator Variables for the Three-Category CSHCN Status (n=973)

	**Untreated Caries (dt)**		**Total Caries Experience (dft)**	
**Variables**	**Estimated AOR (95% CI)**	**p-value**	**Estimated AOR (95% CI)**	**p-value**
(intercept)	0.096 (0.037,0.246)	<0.001*	0.06 (0.030,0.119)	<0.001*
CSHCN Non-Complex ‡ (ref. No)	0.740 (0.525, 1.043)	0.0856	0.913 (0.658, 1.269)	0.589
CSHCN Complex (ref. No)	0.454 (0.224, 0.921)	0.029*	0.481 (0.291, 0.796)	0.004*
Age	1.269 (1.108, 1.454)	<0.001*	1.506 (1.334, 1.700)	<0.001*
Black Race (ref. Non Black)	0.791 (0.624, 1.003)	0.053	0.835 (0.632, 1.104)	0.207
Parent Education > High School (ref. <= HS)	0.792 (0.603, 1.041)	0.094	0.892 (0.676, 1.178)	0.421

†Estimated Odds Ratios with 95% Confidence Interval (CI) and p-values via Wald Chi-Square Tests

***Significance at p<0.05**

‡Additional pairwise comparisons of CSHCH status of complex vs non-complex were completed for untreated caries and caries experience as outcomes (OR = 0.61, 95% CI 0.30, 1.25, p=0.181 and OR = 0.53, 95% CI 0.29, 0.97, p=0.038) respectively

© 2023 Ronis SD et al. *JAMA Network Open.*
