# Supplementary material for: Dental Caries in Medicaid-Insured Preschool Children With or Without Special Health Care Needs in Northeast Ohio
Source: JAMA Netw Open. 2023 Feb 28;6(2):e230999. doi: 10.1001/jamanetworkopen.2023.0999 (PMC9975899; doi:10.1001/jamanetworkopen.2023.0999)
Supplement: Supplement 2. — Data Sharing Statement [file jamanetwopen-e230999-s002.pdf]

Data Sharing Statement

Ronis. Dental Caries in Medicaid-Insured Preschool Children With or Without Special Health Care Needs in Northeast Ohio. *JAMA Netw Open*. Published February 28, 2023. doi:10.1001/jamanetworkopen.2023.0999

Data
Data available: No
